# Cross-cultural communication in a women’s health service: a mixed-methods evaluation

**DOI:** 10.1108/IJHCQA-11-2025-0187

**Published:** 2026-06-15

**Authors:** Georgia Griffin, Safa Ghannam, Yun Su Lin, Mee Mee Zaw, Mahzad Mahdavisharif, Siu Ng, Thi Kim Anh Nguyen, Natalie Williams, Nasrin Javid, Jane Warland, Zoe Bradfield

**Affiliations:** Curtin School of Nursing, Curtin University, Bentley, Australia; Department of Nursing and Midwifery Education and Research, Women and Newborn Health Service, Subiaco, Australia; School of Nursing and Midwifery, College of Health Medicine and Wellbeing, The University of Newcastle, Callaghan, Australia; Adelaide University, Adelaide, Australia

**Keywords:** Communication, Communication barriers, Cultural safety, Limited English proficiency, Medical interpreting, Women’s health services

## Abstract

**Purpose:**

Effective communication is critical to safety and quality in healthcare. Women with limited English proficiency face barriers to quality communication in anglophone settings, perpetuating health inequities. To inform evidence-based strategies, this study aimed to evaluate and explore women’s experiences of cross-cultural communication within a women’s health service.

**Design/methodology/approach:**

Women accessing maternity or gynaecology care at a women’s health service in Western Australia were eligible to participate if they had accessed interpretation support in Arabic, Burmese, Farsi, Mandarin or Vietnamese. A total of 68 women completed a cross-sectional questionnaire; 15 women participated in a semi-structured telephone interview. Quantitative and qualitative data underwent descriptive statistical and multilingual reflexive thematic analyses respectively.

**Findings:**

Participants reported diverse language preferences and skills, most frequently speaking Mandarin, Arabic and Vietnamese. At their most recent visit, most recalled an in-person interpreter present speaking their preferred language. However, less than half were provided with language-concordant written health information resources. Three themes – the role of the interpreter, navigating the health service with uncertainty and a foundation of trust – explore facets of women’s experiences, linked by an overarching theme: health is too important not to understand.

**Originality/value:**

Themes reflect key systemic gaps in the provision of safe quality care for women with limited English proficiency, highlighting the need for proactive service-wide approaches to cross-cultural communication encompassing but not exclusive to clinical encounters. Improved integration of interpretation services, language-concordant information resources and language-concordant support for service navigation are recommended.

## Introduction

Globally, healthcare settings are increasingly culturally and linguistically diverse (Minnican and O’Toole, 2020; Ogbogu *et al*., 2022). Australia is a culturally and linguistically diverse country with a rapidly growing migrant population (United Nations, 2024). International migrants comprised 30.4% of the Australian population in 2024, a notable increase from 23.3% in 1990 (United Nations, 2024). In this context, women from culturally and linguistically diverse (CALD) backgrounds, inclusive of migrants and those with limited English proficiency (LEP), are identified as a priority population in national and state health policies (Department of Health, 2018; Western Australia Department of Health, 2019).

Despite being a priority population, health disparities persist between migrant and Australian-born women (Khatri and Assefa, 2022; Navodani *et al*., 2019; Yelland *et al*., 2015). Women with LEP face intersectional challenges related to language, gendered cultural values and limited health literacy, contributing to disengagement, dissatisfaction with care, reduced involvement in decision-making and autonomy, and poorer health outcomes overall (Hughson *et al*., 2018; Mengesha *et al*., 2017; Yelland *et al*., 2015). A notable Australian study reported that, compared to their Australian-born counterparts, women from non-English speaking backgrounds were more likely to be dissatisfied with perinatal care, feel uninformed and that their preferences were overlooked (Yelland *et al*., 2015). Despite state-wide maternity reforms, little improvement in satisfaction among women with LEP was reported, attributed to a persistent lack of tailored services and inadequate use of interpretation services (Yelland *et al*., 2015).

Language barriers are recognised as impeding effective communication and thereby consumer safety (van Rosse *et al*., 2016). The term cross-cultural communication highlights the cultural lenses that influence communication between people and organisations with different cultures (Minnican and O’Toole, 2020). A rapid review of culturally responsive communication in Australian healthcare settings (Minnican and O’Toole, 2020) has highlighted how culturally responsive communication can result in improved consumer adherence to recommended treatment and improved consumer retention and understanding of health information. Culturally responsive communication was also found to improve consumer and healthcare provider (HCP) satisfaction and consumer health overall (Minnican and O’Toole, 2020).

Professional interpretation can enhance communication with HCPs and improve patient comprehension in hospital settings, compared with interpretation via family members or untrained bilingual staff (van Lent *et al*., 2025; Yeheskel and Rawal, 2019). International systematic reviews have reported improved informed consent processes and follow-up care, and lower readmission rates, with in-person and video modalities preferred by consumers (Rajiv *et al*., 2021; Yeheskel and Rawal, 2019). In maternity settings in Switzerland (Origlia Ikhilor *et al*., 2019) and the United States (Sudhinaraset *et al*., 2023), insufficient access to interpreters has been identified as impeding informed consent and the provision of quality care responsive to women’s needs and expectations. An ethnographic study of communication challenges for immigrant women in rural Canada further found that, while interpreters were necessary to facilitate verbal communication, maternity care providers lacked a strong interpreter infrastructure to draw upon (Higginbottom *et al*., 2015). In the absence of access to interpreters, care providers attempted to adapt communication barriers as they arose, such as through non-verbal communication including hand gestures.

Communication challenges faced by women with LEP extend beyond inadequate access to interpreters (Higginbottom *et al*., 2015; Mengesha *et al*., 2018). Cultural discordance and trauma have been identified as impeding communication between maternity care providers and immigrant women in rural Canada (Higginbottom *et al*., 2015). In Australian and Canadian maternity and sexual and reproductive health (SRH) settings, facilitators of communication with migrant women were identified as trust between healthcare providers and women, confidentiality, adequate time for discussion, female clinicians and interpreters, along with adequate interpreter health knowledge and professionalism (Hawkey *et al*., 2022; Higginbottom *et al*., 2015; Mengesha *et al*., 2018). Women have also expressed preferences for receiving SRH information through multiple modalities, including verbal communication with HCPs and written materials, in primary care settings (Hawkey *et al*., 2022).

Overall, evidence suggests that women with LEP face numerous intersecting challenges to effective communication in maternity and primary SRH settings. When communication is ineffective, poor health outcomes and health inequity are perpetuated (Rajiv *et al*., 2021). To improve health outcomes, insight into cross-cultural communication experiences of people with LEP in anglophone hospital settings is needed (Yeheskel and Rawal, 2019). This study aimed to explore and evaluate the cross-cultural communication experiences of women who communicate with interpreter assistance at a women’s health service.

## Methods

### Methodology

An exploratory mixed-methods design underpinned by a pragmatic paradigm was selected because it enables a rigorous flexible approach to exploration of complex phenomenon such as cross-cultural communication (Biesta, 2010; McBride *et al*., 2019). Phase One comprised an exploratory descriptive cross-sectional questionnaire designed to capture preliminary insights into women’s experiences and perspectives of cross-cultural communication. In Phase Two, a qualitative approach employing reflexive thematic analysis (RTA) was selected, well-suited to inductive exploration of participant experiences (Braun and Clarke, 2021a). Culturally responsive aspects of project design have been reported elsewhere (Griffin *et al.*, 2023, 2024).

### Research setting

This study was conducted in Western Australia (WA), the largest state in Australia, in the geographically isolated, multicultural capital city of Perth (McDonell *et al*., 2024; Office of Multicultural Interests, n.d.). As of the 2021 Australian Census, 32.2% of Western Australians were born outside of Australia (Office of Multicultural Interests, n.d.). A language or languages other than English were spoken at home by 18.7% of Western Australians (Office of Multicultural Interests, n.d.).

The study was set within a women’s health service providing maternity and gynaecological care, which included two hospital sites, one birth centre and a community homebirth program. Women may be referred to the health service for tertiary care from across WA. Whilst annual monetary expenditure on interpretation and translation is reported by language group, the number of women who access these services is not routinely reported. At both hospital sites, interpretation and translation services are coordinated by a single Language Services Department. Interpreters may be hospital employees or booked via external contracted organisations. Provision of an interpreter may be prompted by the woman’s request, a hospital employee identifying a need for an interpreter or a documented need for an interpreter on the woman’s referral letter or in her medical record. Consequently, interpreters may be booked in advance or when a woman presents to the service. At the time of study design, the most frequent languages for which interpreters were booked were Arabic, Burmese, Farsi, Mandarin and Vietnamese, largely reflecting common language groups in WA with high proportions of LEP (Office of Multicultural Interests, n.d.).

### Participant recruitment and sampling

Women were eligible to participate if they had accessed gynaecological or maternity care at the health service within two years of study participation and had utilised interpreter services arranged by the health service in one of five languages: Arabic, Burmese, Farsi, Mandarin or Vietnamese.

Non-probability convenience sampling was employed, appropriate to research with traditionally hard-to-reach populations (Raifman *et al*., 2022). For Phase One, as a cross-sectional descriptive questionnaire was employed, no statistical calculations of sample size were required (Wang and Cheng, 2020). For Phase Two, consistent with the pragmatic underpinnings of the study and Braun and Clarke’s (2021b) approach to RTA, adequacy of the sample was considered in relation to the richness and complexity of the dataset.

Multilingual flyers were disseminated throughout health service waiting areas, community-based organisations and social media groups. Flyers contained a QR code linked to a language-concordant (translated) participant information sheet and online questionnaire portal hosted on REDCap (Harris *et al*., 2019).

Participants could choose to participate in the study by completing an anonymous questionnaire and/or participating in a semi-structured telephone interview. The questionnaire could be completed online or by telephone with a language-concordant bicultural research assistant. Some chose to only participate in an interview. All recruitment and data collection materials were available in Arabic, Burmese, Farsi, English, Mandarin and Vietnamese. Questionnaire participants confirmed their eligibility to participate and provided consent with a “hurdle” item prior to commencing the questionnaire. A consent form was read out to interview participants in their preferred language, following which they provided verbal consent.

### Data collection

A multilingual team of clinician researchers and bicultural research assistants, inclusive of individuals with consumer and professional interpreting experience, developed the questionnaire and interview guide. The tools were refined through a forward and back translation process to ensure meaning was retained across translations. The questionnaire had 29 items: demographic characteristics, single-response, multiple-choice and three open-ended items. The interview guide comprised eight questions to allow further exploration of participants’ experiences. The order of questions was flexible and prompts such as “can you say more about that” facilitated exploration of participant responses. The English language data collection tools are available in the Supplementary Material.

All interviews were conducted by a language-concordant bicultural research assistant. Six team members conducted the interviews, including MMZ, SG, SN, TKAN and GG. Some participants spoke both English and their primary language during the interview at their own preference. Eight interviews were conducted with the addition of an English-speaking-only co-interviewer with clinical experience. This decision was made on review of preliminary interview data because the English-speaking-only interviewer’s clinical experience facilitated further exploration of participants’ experiences. These interviews were conducted in the participant’s primary language and English. Data were collected from July 2023 to August 2024.

### Data analysis

#### Quantitative analysis

Multilingual questionnaire responses planned for quantitative analysis, such as religion, were translated by bicultural research assistants within the team. English data were then entered into SPSS Statistics version 29 (IBM Corp, 2023) for descriptive statistical analysis. Frequencies and percentages were calculated, excluding missing responses.

#### Qualitative analysis

Audio-recorded interviews were transcribed verbatim. Interview and qualitative questionnaire data were translated into English by professional interpreters external to the research team. The team members who conducted the interviews undertook an adapted approach to RTA of a multilingual dataset, moving between the source language and English-language translations. RTA was conducted following Braun and Clarke’s six phases (Braun and Clarke, 2021a), including familiarisation with the dataset; coding; initial theme generation; theme development and review; refinement, definition and naming of themes; and writing up (Braun and Clarke, 2021a). Positionality statements are available in the Supplementary Material. Synthesised member checking was conducted by four Arabic-speaking interview participants to support interpretation of preliminary findings, thereby enhancing trustworthiness (Birt *et al*., 2016). Themes and subthemes were then refined. For analysis, questionnaire participants were allocated a participant number, such as P1. Interview participants could suggest a pseudonym or have one allocated.

### Ethical considerations

Careful consideration was given to language, culture, literacy and power differentials between the research team and eligible women. A multilingual multicultural research team, inclusive of interpreter, consumer and clinician experience, was brought together to enable a culturally responsive approach to the research. The study received approval from the WA Health Human Research Ethics Committee (HREC) (RGS5315) and reciprocal approval from Curtin University HREC (2022-0508).

## Results

In total, 68 women completed the questionnaire, 14 by telephone and 54 online. A total of 15 women participated in a semi-structured telephone interview ranging from 4 to 71 min (mean 34 min). Thirteen participants consented to being audio-recorded. Notes were taken for two participants who did not consent to recording, and during telephone questionnaires and interviews to facilitate later interpretation of the data. Interview participants included women who did not complete a questionnaire.

### Participant characteristics

#### Questionnaire participants

Demographic characteristics of questionnaire and interview participants are presented in Table 1. Participants demonstrated substantial heterogeneity across length of residence in Australia, country of birth, self-reported ethnicity, primary language and religion. Of 68 questionnaire participants, 47 (69.1%) accessed maternity care and 21 (30.9%) accessed gynaecology care. Participants were aged 23–67 years (mean 38.9 years, median 36 years). They had lived in Australia between eight months and 25 years (mean 7.7 years, median 6 years), and were born in one of 15 countries, most frequently China (*n* = 21, 31.3%) or Vietnam (*n* = 11, 16.4%). When asked to describe their ethnicity, participants nominated 28 different responses, reflecting the diversity within the five language groups. Both ethnicities and languages are reported as described by participants. Common ethnicities included Han (*n* = 14, 21.9%) and Vietnamese or Viet Nam (*n* = 6, 9.4%). Most participants reported no religion (*n* = 24, 37.5%) or described themselves as Muslim (*n* = 22, 34.4%). When asked their primary language, 14 languages were reported, most frequently Mandarin (*n* = 19, 28.4%), Arabic (*n* = 13, 19.4%) or Vietnamese (*n* = 11, 16.4%). Five participants (7.5%) reported two primary languages.

**Table 1:** Participant demographic characteristics and reasons for attending the health service

	Questionnaire *n* (%)^a^	Interview *n* (%)^a^
Reason for attending the health service (N = 68)*		
Pregnancy care and complications	29 (42.6)	5 (33.3)
Intrapartum care	20 (29.4)	4 (26.7)
General gynaecology	13 (19.1)	3 (20.0)
Urological gynaecology	7 (10.3)	3 (20.0)
Postnatal care	6 (8.8)	0 (0.0)
Gynaecological cancer	3 (4.4)	1 (6.7)
Pregnancy loss	1 (1.5)	0 (0.0)
Other	4 (5.9)	1 (6.7)
*Age in years (N = 55)*		
≤25	3 (5.5)	0 (0.0)
26–35	21 (38.2)	4 (26.7)
36–45	19 (34.5)	5 (33.3)
46–55	6 (10.9)	2 (13.3)
56–65	4 (7.3)	2 (13.3)
≥66	2 (3.6)	2 (13.3)
*Number of years of residence in Australia (N = 68)*		
≤5	32 (47.1)	4 (26.7)
6–10	17 (25.0)	6 (40.0)
11–15	14 (20.6)	2 (13.3)
≥16	5 (7.4)	3 (20.0)
*Country of birth (N = 67)*		
China	21 (31.3)	5 (33.3)
Vietnam	11 (16.4)	1 (6.7)
Iraq	7 (10.4)	3 (20.0)
Taiwan	6 (9.0)	1 (6.7)
Iran	6 (9.0)	0 (0.0)
Myanmar	5 (7.5)	1 (6.7)
Malaysia	2 (3.0)	0 (0.0)
Afghanistan	2 (3.0)	0 (0.0)
Other	7 (10.4)	4 (26.7)
*Ethnicity* ^*†*^ *(N = 64)*		
Han	14 (21.9)	1 (7.1)
Vietnamese or Viet Nam	6 (9.4)	1 (7.1)
Iranian	5 (7.8)	0 (0.0)
Arab	4 (6.3)	4 (28.6)
Chinese or China	4 (6.3)	1 (7.1)
Kinh	4 (6.3)	0 (0.0)
Taiwan	3 (4.7)	0 (0.0)
Chin	2 (3.1)	0 (0.0)
Ethnic Han	2 (3.1)	0 (0.0)
Iraqi	2 (3.1)	1 (7.1)
Other	18 (28.1)	6 (42.9)
*Religion (N = 68)*		
No religion	24 (37.5)	3 (20.0)
Muslim	22 (34.4)	7 (46.7)
Christian	10 (15.6)	3 (20.0)
Buddhist	7 (10.9)	2 (13.3)
Bahai	1 (1.6)	0 (0.0)
Primary language spoken (N = 67)*		
Mandarin	19 (28.4)	6 (40.0)
Arabic	13 (19.4)	7 (46.7)
Vietnamese	11 (16.4)	1 (6.7)
Chinese	8 (11.8)	0 (0.0)
Farsi	7 (10.4)	0 (0.0)
Burmese	3 (4.5)	1 (6.7)
Cantonese	2 (3.0)	1 (6.7)
Chin	2 (3.0)	0 (0.0)
English	2 (3.0)	1 (6.7)
Other	5 (7.5)	0 (0.0)

**Note(s):** *Multiple response item. Percentages reflect the proportion of participants who selected each response option. ^†^Ethnicity is shown using terms used by participants to reflect self-identification rather than standardised classifications

^a^ Valid percentage. Missing data removed

#### Interview participants

From 15 interview participants, eight (53.3%) accessed maternity care and seven (46.7%) accessed gynaecological care. They were aged 30–76 years (mean 46 years, median 38 years). They had lived in Australia for 1 to 26 years (mean 9.1 years, median 6 years) and were born in one of eight countries, most frequently China (*n* = 5, 33.3%) or Iraq (*n* = 3, 20.0%). Interview participants reported eight ethnicities, most frequently Arab (*n* = 4, 28.6%). Most described themselves as Muslim (*n* = 7, 46.7%). Six primary languages were reported, most frequently Arabic (*n* = 7, 46.7%) and Mandarin (*n* = 6, 40.0%). Both questionnaire and interview participant characteristics are presented in Table 1.

### Verbal communication

Questionnaire participants’ perspectives of interpreter-mediated verbal communication are presented in Table 2. At their most recent visit to the study site, interpretation services were most frequently provided in Mandarin (*n* = 22, 34.4%), Arabic (*n* = 13, 20.3%) and Vietnamese (*n* = 10, 15.6%). Equal proportions (48.5% versus 48.5%) of participants recalled that interpretation services were provided because they had requested or because staff had offered. Most (*n* = 67, 98.5%) reported that the interpreter spoke their preferred language. Most (*n* = 67, 98.5%) also reported feeling confident to request an interpreter. Interpretation modality was most frequently in-person (*n* = 62, 91.2%), aligning with participant preferences. Most participants selected in-person interpretation as their preferred modality (*n* = 57, 93.4%). Twenty-two participants (32.4%) recalled that telephone interpretation was provided. Only two participants (3.3%) identified telephone interpretation as their preferred modality.

**Table 2:** Perspectives of interpreter-mediated verbal communication

	Total *n* (%)^a^
*Language spoken by interpreter at most recent visit (N = 64)*	
Mandarin	22 (34.4)
Arabic	13 (20.3)
Vietnamese	10 (15.6)
Farsi	8 (12.5)
Burmese	5 (7.8)
Chinese	5 (7.8)
Dari	1 (1.6)
*Alignment with language preference (N = 68)*	
Interpretation provided in their preferred language	67 (98.5)
Interpretation not provided in their preferred language	1 (1.5)
*Prompt for interpreter services (N = 68)*	
Participant requested	33 (48.5)
Staff initiated	33 (48.5)
Both participant requested and staff initiated	1 (1.5)
Unsure	1 (1.5)
*Confidence to ask for an interpreter (N = 66)*	
Confident	62 (93.9)
Not confident	4 (6.1)
Interpretation modality experienced (N = 68)*	
In-person	62 (91.2)
Telephone	22 (32.4)
Telehealth or video conference	4 (5.9)
iPad application	0 (0.0)
First preference for interpretation modality (N = 61)*	
In-person	57 (93.4)
Telephone	2 (3.3)
Telehealth or video conference	0 (0.0)
iPad application	1 (1.6)
Telephone and iPad application	1 (1.6)
Second preference for interpretation modality (N = 46)*	
In-person	0 (0.0)
Telephone	28 (60.9)
Telehealth of video conference	12 (26.1)
iPad application	5 (10.9)
*Comfortable to communicate with the assistance of an interpreter (N = 68)*	
Comfortable	66 (97.1)
Not comfortable	2 (2.9)
Factors promoting comfort (N = 67)*	
Friendly HCP	44 (65.7)
Friendly interpreter	44 (65.7)
Female HCP	34 (50.7)
Female interpreter	31 (46.3)
Family member, friend or support worker present	24 (35.8)
Same HCP as before	18 (26.9)
Same interpreter as before	15 (22.4)
Other	4 (6.0)

**Note(s):** *Multiple response item. Percentages reflect the proportion of participants who selected each response option

^a^ Valid percentage. Missing data removed

Most participants reported feeling comfortable communicating with the assistance of an interpreter (*n* = 66, 97.1%). Factors that promoted comfort were most frequently a friendly HCP (*n* = 44, 65.7%), a friendly interpreter (*n* = 44, 65.7%) and a female HCP (*n* = 34, 50.7%). Multiple responses could be selected.

### Written communication

Questionnaire participants’ self-rated literacy skills and perspectives of written communication are presented in Table 3. Most participants reported being able to read (*n* = 64, 97.0%) and write (*n* = 62, 95.4%) well or very well in their primary language. Twenty-two participants (34.4%) reported being able to read in English well or very well, and 21 participants (32.3%) reported being able to write in English well or very well. Most participants reported feeling confident to ask for written health information (*n* = 58, 86.6%). However, while 70.6% of participants (*n* = 48) were offered written information in English, only 44.8% (*n* = 30) preferred to receive written information in English. Most participants (*n* = 52, 77.6%) preferred that written information be provided in the language spoken by the interpreter present. Only 42.6% (*n* = 29) were offered written information in that language.

**Table 3:** Self-reported literacy skills and perspectives of written communication

	Total *n* (%)^a^
*Self-rated ability to read in primary language (N = 66)* ^*b*^	
Very well	43 (65.2)
Well	21 (31.8)
Not well	1 (1.5)
Not at all	1 (1.5)
*Self-rated ability to write in primary language (N = 65)* ^*b*^	
Very well	40 (61.5)
Well	22 (33.8)
Not well	1 (1.5)
Not at all	2 (3.1)
*Self-rated ability to read in English (N = 64)* ^*b*^	
Very well	1 (1.6)
Well	21 (32.8)
Not well	36 (56.3)
Not at all	6 (9.4)
*Self-rated ability to write in English (N = 65)* ^*b*^	
Very well	1 (1.5)
Well	20 (30.8)
Not well	36 (55.4)
Not at all	8 (12.3)
*Confidence to ask for written information (N = 67)*	
Yes, confident	58 (86.6)
Not confident	9 (13.4)
Language of written information offered (N = 68)*	
English	48 (70.6)
Language spoken by the interpreter	29 (42.6)
In a different language or languages	1 (1.5)
No information offered	3 (4.4)
Not sure	3 (4.4)
Language preference for written information (N = 67)*	
English	30 (44.8)
In the language spoken by the interpreter	52 (77.6)
In a different language or languages	2 (3.0)
No written information wanted	4 (6.0)

**Note(s):** *Multiple response item. Percentages reflect the proportion of participants who selected each response option

^a^ Valid percentage. Missing data removed

### Exploration of cross-cultural communication experiences

To meet the research aim, to explore the cross-cultural communication experiences of women who communicate with interpreter assistance at a women’s health service, qualitative data from 153 responses to the three open-ended questionnaire items from 62 women and interview transcripts and field notes from 15 interviews underwent RTA. The questionnaire items were: Please say why you asked for an interpreter; Please say why you did or did not feel confident; and Why did you rank [preferred interpreter modality] as your first preference? Interview questions are presented in the Supplementary Material.

In exploring how women experienced communication, one overarching theme, three interrelated themes and eight subthemes were constructed. The overarching theme, *Health is too important not to understand*, underpinned the subsequent themes and subthemes. It illustrated how women expressed a concern that ineffective communication could compromise their health or treatment. Good health and effective care were the priority. Communication occurred in the context of feeling unwell, worried, fearful or tired, which both impeded communication and heightened the perceived impact of miscommunication on their health outcomes.

… there are words that the doctor may say and I would not understand them correctly and would lose important information about my pregnancy (P4^F,M^)

**Figure 1:**
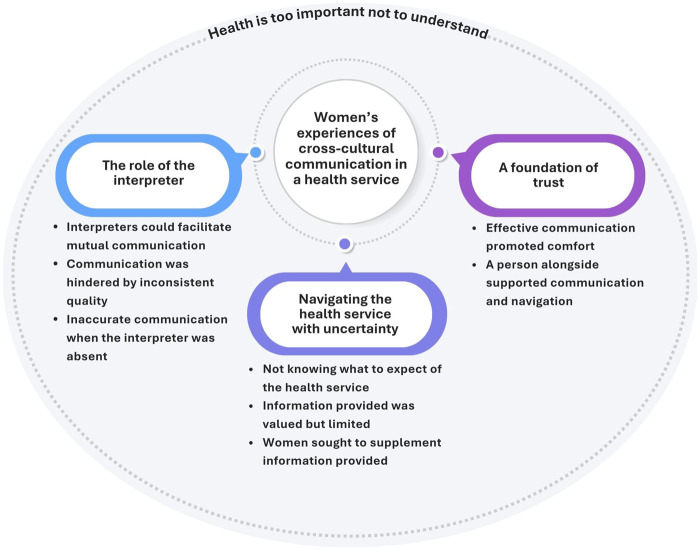
Thematic map of women’s cross-cultural communication experiences

Themes and subthemes are described below with supporting quotes presented in English, followed by a participant number or pseudonym. Superscript letters indicate language group: A for Arabic, B for Burmese, F for Farsi, M for Mandarin and V for Vietnamese. The following superscript letters M and G indicate if the participant accessed maternity or gynaecology care. Original quotes in the source language are provided in the Supplementary Material. A thematic map is presented in Figure 1.

### Theme 1: The role of the interpreter

Interpreters were arranged by the health service at specific points in women’s care, primarily during outpatient appointments or to gain consent for procedures. In person, an interpreter with sound knowledge of medical terminology facilitated mutual communication between women and HCPs. However, when interpreters appeared rushed, disengaged or unsure of health concepts, women were left without understanding of their care. Women attempted to communicate around language barriers when interpreters were not made available, sometimes describing dissatisfaction with communication and/or their care. The following three subthemes explore how interpreters facilitated mutual communication, how inconsistent quality of interpretation hindered communication, and how inaccurate communication occurred when the interpreter was not present.

#### Subtheme 1.1: Interpreters could facilitate mutual communication

Women described a spectrum of English language skills, from being unable to communicate in English to being confident in everyday English but unfamiliar with medical terminology. In this context, women described how interpreters facilitated mutual communication with HCPs by helping them to understand what was being said, explain their concerns and ask questions.

When in-person, if I’m confused with something or I don’t quite understand something, I can ask the interpreter at once and so the interpreter can explain it to me immediately. Therefore, it creates a connection between the interpreter and me (P12^B,M^)

They felt that interpreter-mediated communication was easier, more direct, efficient and friendly. An interpreter present in-person allowed women, interpreters and HCPs to observe each other’s facial expressions and body language, improving the flow of communication. When interpretation was effective, it enhanced women’s confidence in their understanding of their condition or proposed care, from which they could make decisions about their health.

My ability to read and write English is good, and I can do daily things easily by myself … I was not sure whether I would fully understand medical terms in English, and I needed to receive accurate information about the surgery that I needed, from my doctor (P39^F,G^)

#### Subtheme 1.2: Communication was hindered by inconsistent quality

Women recalled an array of barriers influencing the quality of the interpretation provided. They reflected that not all interpreters appeared to have a sound knowledge of medical terminology or health concepts, resulting in misinterpretation and misunderstandings between all parties. One woman expressed concern that some interpreters appeared to add their own opinions to the information they interpreted on behalf of the HCP: “*the translation wasn’t very accurate. Also, when the midwife was explaining things to me and I had some questions, the interpreter would answer using her own experiences”* (Eva^M,M^). Others were concerned that some interpreters appeared rushed or disengaged. This was sometimes associated with prolonged wait times for outpatient appointments, exceeding the length of time interpreters were booked for: *“… many times, interpreters had to leave when their booking time was over, even though my consultation with the medical professionals had not completed”* (P67^M,M^).

If the interpreter was unable to stay, a telephone interpreter was then accessed. However, using telephone interpreters was often associated with technical problems, long wait times and an apparent lack of staff knowledge about how to arrange them. Once connected, the interpretation itself was typically described as less accurate and communication as less direct. Experiences of ineffective interpretation reduced women’s confidence in their understanding of their health and plan of care.

… they asked me to sign the consent form for induction … A male interpreter [was connected by telephone], but the interpreting he did, I think he probably did not know how to interpret some of the professional information, especially the things about labour and delivery … I think I understood more listening to the English. His understanding was probably no better than mine (Helen^M,M^)

#### Subtheme 1.3: Inaccurate communication when the interpreter was absent

When an interpreter was not present, women communicated within the limits of their English language skills and appreciated HCP efforts to speak slowly to try to help them understand. Some asked a friend, family member or support worker present to assist them with communication. Others described using a translation or artificial intelligence phone app, although the translation could be inaccurate, *“… besides using my phone, I didn’t have any other methods [to interpret] … it wasn’t effective. It was quite inconvenient, as it took up a lot of the doctor’s time, and the translation wasn’t always accurate …”* (Kitty^M,M^). Another woman described trying to learn English medical terms in preparation for labour and birth, having been advised that an in-person interpreter would not be made available.

Without an interpreter, women described barriers to understanding what was said, to asking questions and to communicating their concerns. This caused worry and confusion: *“… there wasn’t any interpreter at the [emergency department] … I did not fully express myself … I just, just listen and accept”* (Helen^M,M^). Some disengaged from care: *“since I didn’t understand what she was saying, I just ignored it”* (Khine^B,G^). Others sought alternate avenues to seek healthcare, such as presenting multiple times to the emergency department or contacting a trusted general practitioner (GP). Others consented to care without understanding what was happening,

… [the midwife] said she needed to give me a shot of something, I just, I went a bit lost, didn’t really understand. I just replied. OK, OK. And [she] gave me the shot. Yeah, I did not know what she gave me (Helen^M,M^)

### Theme 2: Navigating the health service with uncertainty

Women described experiences of navigating the health service and their overall care characterised by uncertainty. Expectations of care and its delivery were informed by previous healthcare experiences, norms in their country of origin, and information sought online and from peers. Uncertainty existed regarding their right to an interpreter, when these rights applied and what level of autonomy they had over the interaction. Women valued when information was communicated effectively about what to expect and treatment options; however, both the information content and how it was communicated could be inadequate. The following three subthemes explore women’s experiences of not knowing what to expect of the health service, valued but limited information provided, and how women sought to supplement the information provided.

#### Subtheme 2.1: Not knowing what to expect of the health service

Many women described uncertainty about what to expect when accessing care. This could include the purpose of each outpatient appointment, what type of HCP they would see and treatment options: *“… I just take my referral from my GP and to see the hospital … without any knowledge about what we will do next, yeah. So that’s make me a little bit worried …”* (Lily^M,M^). Women accessing gynaecological care specifically described uncertainty about how long to expect to wait for test results or surgery, and about the frequency and method of follow-up appointments. There was uncertainty about potential out-of-pocket costs: “*[I was]* c*oncerned that [an interpreter] might involve fees”* (P40^M,G^). Many women also expressed uncertainty about whether and when they had a right to an interpreter, whether they could choose when they wanted an interpreter to be present and not present, or if they could request a specific interpreter. Misconceptions were reported, such as not being permitted an interpreter for telephone interactions or being ineligible to access continuity of midwifery care models. Repeated instances were described in which interpreters were not made available during telephone consultations, when presenting for emergency care and in labour and birth: “*I asked once [for an interpreter], and they told me that we can’t provide you with this service”* (Dalal^A,G^). Women found it difficult to advocate for themselves when they were unsure what was normal and what choices they had access to in Australia.

#### Subtheme 2.2: Information provided was valued but limited

Women described limited verbal and written information provided about what to expect of their care. During appointments, women valued being told what may be discussed during their next appointment or when to expect to be contacted next. Outside of appointments, information advising women about their next appointment or surgery was communicated in English by text message, email or letter.

… it’s not good, because I have to give it to someone to read it to me … this is an Arab who needs an interpreter [so] send them the letter … [in] Arabic so that they don’t miss the appointment … (Dalal^A,G^)

Typically, appointment notifications were restricted to a date, time and location. However, women wanted more practical information to allow them to plan appropriately, such as if an interpreter had been booked and a hospital map or directions, “*… provide information on whom I need to see at each appointment and roughly how long the whole process will take”* (Mavis^M,M^).

Some women depended on a family member or support worker to explain information. Others were confident in their English literacy skills and used an app to translate as needed. Their comprehension could be hindered, however, by tiredness and inaccuracies in web-based translations.

… the hospital gave me a booklet. It contains details about how things develop week by week and which tests are needed … I just felt very tired when I was pregnant … since English, after all, is not my first language, I wasn’t much motivated to read it anyway. (Eva^M,M^)

#### Subtheme 2.3: Women sought to supplement information provided

Some women described speaking with others from similar cultural backgrounds or searching language-concordant online forums and social media apps to gauge what was normal with respect to their care or treatment. However, they expressed uncertainty about the reliability and accuracy of this information. They valued being able to check this information with an HCP at the health service or a trusted GP. They also valued websites identified by HCPs as reliable, *“… if there some … Website that midwife give to us, or some book that midwife give to me … I can trust to follow it”* (Lily^M,M^). Others were not confident or interested in searching for information.

Online antenatal education classes offered by the health service were attended by some women; however, comprehension could be impeded by fast-paced English and the absence of interpreter support. Some described instead seeking language-concordant information online, such as Australian Chinese language antenatal classes, *“… both Chinese and English were provided [in the external class], and she … offered more information for me to understand how the delivery process unfolds in Australia, and what the environment was like in an Australian hospital*” (Helen^M,M^).

### Theme 3: A foundation of trust

Women approached the health service and HCPs with trust associated with HCPs’ perceived expertise. Many stated that trust was enhanced through good health outcomes, effective communication and acts of cultural safety. Having a trusted person by their side helped women navigate the health service with confidence, and interpreters could be viewed by women as having a support role. Both HCP and interpreter continuity were valued if they had built trust with the woman; continuity of either was rarely encountered. The following two subthemes explore women’s experiences of how effective communication promoted comfort and having a familiar person to promote communication and service navigation.

#### Subtheme 3.1: Effective communication promoted comfort

Women described trust in HCPs and the health service associated with a perception of HCPs’ expertise, *“trust the services provided in hospitals”* (P66^M,M^). HCPs could build upon this foundation of trust through good health outcomes and effective communication. Effective communication was enacted by HCPs by providing an interpreter, providing language-concordant written information and speaking women’s preferred language. Women also valued HCPs’ professionalism and a friendly and welcoming manner: *“the doctors here are very attentive, professional, and patient. So I feel comfortable communicating with them”* (Kitty^M,M^).

Seeing the same HCP was valued because continuity over multiple interactions was perceived to enable the HCP to learn how to communicate effectively with that woman, *“… if we can just meet the similar [same] midwife, that would be really helpful … he will just know how to communicate with me, or [if they need] to speak a little bit slow down so I quite understand it …”* (Lily^M,M^). However, continuity of care provider was rare. Additionally, as women became more familiar with how care was delivered, they became more comfortable, for example, “*… they also asked with care, they asked every time they met me, that if I was mentally well? Did I need any support? Did I feel safe? … At first, I felt a little strange. But then I felt more assured*” (Jade^V,M^).

Some women described a loss of trust when communication broke down, interpreters were not provided, or their health needs were not met. One woman described a pre-existing distrust of HCPs due to a previous negative experience at a different hospital during which poor care had been blamed on her perceived lack of English language skills. She requested an interpreter at this health service to protect herself, *“… I do not want something to happen to me and then [the HCPs] blame me for their inexperience and say that you do not understand the language”* (P61^F,M^).

#### Subtheme 3.2: A person alongside supported communication and navigation

Having a person to stand by their side throughout their care was valued by some women and desired by others: *“if I were to suggest an improvement, it would be having someone accompany me when I go for things like blood tests or check-ups in other departments”* (Kitty^M,M^). For some, female in-person interpreters could fulfil this role, while for others, a support person, such as a partner, family member or support worker could accompany them. Having someone by their side was a source of comfort and confidence for women. Support people were described as assisting women with communication alongside interpreters, and for some women, were preferred to a hospital-provided interpreter: *“[My support workers] share their experiences and what they know with me, offering guidance and helping me”* (Khine^B,G^).

Women valued someone waiting with them for and between appointments, helping them to find different locations within the hospital and offering advice from their personal experiences. This could include an interpreter, support worker or family member. For this reason, some women valued having an interpreter provided in-person rather than by telephone. If an interpreter was viewed as professional and skilled, and offered support and advice, trust could be established.

… I really liked [the interpreter] because she … had two babies and she gave me lots of the experience what she said and lots of the details about that hospital, to tell [me] … what to do, and also let me [be] a little bit clear about if I meet this situation, what I maybe say. So she gave me lots of information that’s really helpful (Lily^M,M^)

Continuity of interpreter was valued in these instances, although rare.

## Discussion

This study offers insights into women’s experiences of cross-cultural communication in an Australian women’s health service. Quantitative findings reveal the diversity amongst women who use interpreter services and identify unmet communication and information needs. Qualitative findings highlight the key role of interpretation in facilitating effective communication, women’s uncertainty navigating the health service and the value of having someone to support communication and navigation. Overall, the study highlights the need for a proactive service-wide approach to communication. Key systemic gaps are explored in the following discussion, highlighting inconsistent integration of interpretation services, a lack of language-concordant resources to promote informed decision-making and a need for support with system navigation.

Our findings affirm the key role of professional interpretation in cross-cultural communication. However, while most participants reported that an in-person interpreter was provided in their preferred language at their most recent health service visit, interpretation services were not provided consistently and interpretation quality varied. Inadequate interpretation services are a recurrent theme in maternity and broader health services, identified in both Australian (Hughson *et al*., 2018; Mengesha *et al*., 2016; Minnican and O’Toole, 2020; Yelland *et al*., 2016) and international literature (Allegri *et al*., 2025; Origlia Ikhilor *et al*., 2019; Phillimore, 2015). The value of professional interpretation to facilitate communication in hospital settings has been well established (Kwan *et al*., 2023; Rajiv *et al*., 2021), yet systemic barriers persist. One recent Australian mixed-methods study investigating HCP perspectives of interpreter access during the COVID-19 pandemic identified limited availability of in-person interpreters, long wait times for telephone interpreters and an inflexible booking system as barriers (Tang *et al*., 2024).

These barriers reflect a broader systemic issue; when interpreters are engaged through external agency-based casual employment models rather than employed within health services, it limits health services’ abilities to improve the availability, accessibility and quality of interpretation services. Training and support for interpreters have been recommended to reduce variability in interpreter quality, particularly for clinical areas requiring nuanced communication such as women’s reproductive health (Khoong and Fernandez, 2021); however, health service governance over support and training provided to external interpreters is limited in the current employment model. This approach may also limit the ability of health services to systematically collect data to monitor, evaluate and improve interpretation services (Kwan *et al*., 2023), and diminishes recognition of interpreters as an essential workforce in the healthcare system. Further research is recommended to identify economic, financial and organisational factors underpinning the current employment model towards exploring sustainable models to better integrate professional interpreters into health services and to evaluate the impact of these models on clinical and economic outcomes.

Our study findings show that interpretation services alone were oftentimes insufficient to meet the communication needs of women with LEP. Participants highlighted a lack of language-concordant resources provided to support verbal communication, promote informed decision-making and facilitate health service navigation. Less than half of the participants were offered written information in their preferred language. These findings may reflect a lack of easily accessible multilingual information resources, aligning with the need for the improved provision of translated health resources reported in a scoping review of international research into the patient experience of people with LEP (Yeheskel and Rawal, 2019). Similarly, a qualitative Australian study investigating strategies to address health literacy issues in the provision of maternity care to culturally and linguistically diverse women identified a need for improved provision of culturally and linguistically appropriate eHealth resources as adjuncts to verbal communication (Hughson *et al*., 2018). The authors specifically recommended a hospital-endorsed multilingual health information app.

When exploring pathways to improve availability and provision of multilingual resources, translating materials designed for English-proficient audiences can limit their relevance and acceptability (Taira *et al*., 2019). Our findings show that women with LEP may have specific information needs associated with navigating an unfamiliar health system which may not be met through translation alone. The importance of participatory approaches in the design and implementation of information resources responsive to the needs of culturally and linguistically diverse populations has been emphasised in maternity and health promotion settings (Gray *et al*., 2024; Hughson *et al*., 2018). Adopting a co-design approach more broadly within hospital settings is warranted to ensure women have access to language-concordant resources responsive to the diversity of their information needs.

Our study participants described uncertainty in navigating the health service, exacerbated by English-language hospital correspondence lacking information content to support service navigation. In their scoping review of the patient experience, Yeheskel and Rawal (2019) similarly reported appointment letters and forms issued in English only, associating this English-language communication with poor appointment attendance. A content analysis of acute care hospital websites located in one state in the United States (Graves *et al*., 2020) further found that only 10.8% provided translated content and while 81.7% of websites identified the availability of interpretation services, this information could only be accessed after navigating through multiple English-language webpages. These findings demonstrate the challenges that people with LEP can face in supplementing inadequate hospital communication by searching online. The study authors recommended that hospitals improve their online communication strategies to meet the needs of linguistically diverse communities (Graves *et al*., 2020). Our findings highlight the need for proactive language-concordant communication strategies beyond clinical interactions to effectively support service navigation by women with LEP.

Finally, our study reports a need for in-person support for service navigation, with women often highlighting advice and support provided by an interpreter. Service navigation, however, is beyond the scope of professional interpreters in Australia (Australian Institute of Interpreters and Translators Inc, 2012). The Australian Institute of Interpreters and Translators Code of Ethics and Code of Conduct outlines the role of interpreters as facilitators of communication through message transfer, precluding their involvement in advocacy, guidance or advice (Australian Institute of Interpreters and Translators Inc, 2012). Despite this, participants described instances of interpreters acting outside this scope, with some appreciating this and others not. This suggests a gap in advocacy and support for service navigation. Previous research has explored language-concordant health navigator or cultural broker roles in Australia (Riggs *et al*., 2017) and internationally (DiMeo *et al*., 2023). DiMeo *et al*. (2023), in their critical review of cultural broker roles in pregnancy care, emphasise the importance of role clarity and a supportive ecosystem for the role to be successful. Our findings suggest there is a role for navigators; however, further research is needed into the sustainable implementation and impact of language-concordant health navigator roles within hospital settings.

### Strengths and limitations

This study provides much needed data on consumer perspectives of cross-cultural communication. Unlike previous research that identified HCP skills and training as an area for improvement (Minnican and O’Toole, 2020), participants in this study reflected little on this aspect of their care. This may be because the study was conducted by the health service that they had received or were continuing to receive care through. The research team was not involved in participants’ clinical care, and participants were informed that participation would not impact their care; however, this power dynamic may have affected the findings. Because the number of women who accessed interpreter services annually was not reported at the study site, it was not possible to determine the representativeness of the sample size. The small sample size and potential for recall bias may be considered limitations of this study; however, the use of a mixed-methods design is a strength (McBride *et al*., 2019). Triangulation of quantitative and qualitative data allowed for exploration of key aspects of communication and provided insights into aspects of women’s experiences that may not have been captured by one method alone. Throughout the design and conduct of this study, an emphasis was placed on cultural responsiveness and language concordance, exemplified through the crucial role of bicultural bilingual research assistants in tool design, data collection and analysis. It is possible, however, that nuances in women’s experiences were missed due to language or cultural discordance.

## Conclusion

In an increasingly multicultural world, cross-cultural communication requires a proactive whole-of-health service approach to meet the communication and service navigation needs of women with LEP. This study shows that women’s experiences of communication extend beyond clinical interactions; however, support for communication largely did not. Within clinical interactions, the provision and quality of interpretation services were variable. Planning and resourcing to support effective cross-cultural communication, particularly with respect to the integration of interpretation services and language-concordant support for service navigation, must be key considerations for service and clinical area leads and policymakers in the delivery of healthcare.

## Supplementary Material

Data supplement 1

## Data Availability

The data that support the findings of this study are available on reasonable request from the corresponding author, GG. The data are not publicly available due to ethical restrictions.

## Notes

The supplementary material for this article can be found online.
