# Supplementary material for: Cross-cultural communication in a women’s health service: a mixed-methods evaluation
Source: Int J Health Care Qual Assur. 2026 Jun 15:1–20. doi: 10.1108/IJHCQA-11-2025-0187 (PMC13261826; doi:10.1108/IJHCQA-11-2025-0187)
Supplement: Data supplement 1 [file ijhcqa-11-2025-0187_suppl1.docx]

**Supplementary material**

**Cross-Cultural Communication Project Survey Tool**

*The English language survey tool presented below was translated for online and telephone data collection into Arabic, Burmese, Farsi, Mandarin and Vietnamese.*

First, please confirm that you have read the Participant Information Form or had the Participation Form read to you, that you received maternity or gynaecology care at [hospital site 1] or [hospital site 2] and consent to participating in this survey. You can find a copy of the Participant Information Form at this link.

- Yes, I have read the Participant Information Form and I understand the purposes, procedures and risks of the research described in the project. I understand that I am free to withdraw up until submission of my survey responses, and that withdrawing will not affect my future health care. I freely consent to participate in this research project as described.
- Yes, I received maternity or gynaecology care at [hospital site 1] and/or [hospital site 2].

These next questions are some information about you.

1. What is your age? _____________________
2. What did you attend [the health service] for?

- Urological gynaecology (problems with your bladder)
- General gynaecology (problems with your uterus/ovaries/vagina that are not related to cancer)
- Gynaecological cancer
- Antenatal care (clinics)
- Pregnancy complications (e.g. Maternal Foetal Assessment Unit/Emergency Centre)
- Labour and birth
- Postnatal care
- Pregnancy loss or complications (less than 20 weeks)
- Pregnancy loss (more than 20 weeks)
- Other: ________________

1. What ethnicity do you identify as? ______________
2. What is your country of birth? ________________
3. How many years have you lived in Australia? _________________
4. What is your religion? ________________________
5. What is the primary language you speak? ______________
6. How well can you read in your primary language?

- Not at all
- Not well
- Well
- Very well

1. How well can you write in your primary language?

- Not at all
- Not well
- Well
- Very well

1. How well can you read in English?

- Not at all
- Not well
- Well
- Very well

1. How well can you write in English?

- Not at all
- Not well
- Well
- Very well

1. In your most recent visit, what language did the interpreter speak? _____________
2. Was this your preferred language?

- Yes
- No

1. Did you ask for an interpreter or was an interpreter offered by staff?

- Asked *(Branching logic to Q15)*
- Offered *(Branching logic to Q16)*
- Not sure *(Branching logic to Q16)*

1. Please say why you asked for an interpreter
2. Did you feel confident to ask for an interpreter?

- Yes
- No

1. Please say why you did or did not feel confident
2. What ways have you communicated with an interpreter at this hospital?

- In-person
- Telephone
- Video conference/Telehealth
- iPAD app

1. Please rank interpreter type in order of preference:

- In-person
- Telephone
- Video conference/Telehealth
- iPAD app

1. Why did you rank [*selection*] as your first preference?
2. Did you feel comfortable communicating with the healthcare provider (midwife, nurse, doctor, physiotherapist etc) through the interpreter?

- Yes
- No

1. What makes you feel comfortable when communicating with a healthcare provider?

- Female healthcare provider
- Female interpreter
- The same healthcare provider I’ve seen before
- The same interpreter I’ve seen before
- Friendly healthcare provider
- Friendly interpreter
- Family member, friend or support worker present
- Other: __________________________________________

1. Were you offered written information in English or your primary language?

- Yes, in English
- Yes, in [language]
- No
- Not sure

1. Would you like to be offered written information?

- Yes, in English
- Yes, in [language]
- No

1. Do you feel confident to ask for written information?

- Yes
- No

1. Are you aware of the [health service] Library as a place to access written health in information in [language]?

- Yes, I already use the library
- Yes but I don’t use the library
- No

1. Do you use technology to communicate about or find information about your [pregnancy/condition]?

- Yes, I use a phone app: _________
- Yes, I use a computer
- No

1. Do you use the internet to find information about your [pregnancy/condition]?

- Yes, I already do *(Branching logic to Q28a)*
- No but I would like to *(Branching logic to Q28b)*
- No, I don’t want to *(Branching logic to Q29)*

28a. If you use the internet, how do you make sense of information that is not in [*primary* language]?

- Use a translation app or website (e.g. Google Translate)
- Ask a friend/family member/child
- Look at videos or pictures
- Only access information in my primary language
- Only access information from my country of origin
- Other: _______________________

28b. If you would like to use the internet to find information about your [pregnancy/condition], what is stopping you?

- Don’t have technology skills
- Don’t know where to look
- Not sure what are reliable websites
- Find it difficult to translate information
- Don’t have access to the internet
- Too busy
- Other: _______________________

1. This is the end of the survey. Once I submit your response, you will no longer be able to withdraw. Do you consent for your responses to be submitted?

- Yes

**Cross-Cultural Communication Project Interview Guide**

*The English language interview guide was translated into Arabic, Burmese, Farsi, Mandarin and Vietnamese.*

**Demographic questions**

1. What is your age? _____________________
2. What did you attend [the health service] for?

- Urological gynaecology (problems with your bladder)
- General gynaecology (problems with your uterus/ovaries/vagina that are not related to cancer)
- Gynaecological cancer
- Antenatal care (clinics)
- Pregnancy complications (e.g. Maternal Foetal Assessment Unit/Emergency Centre)
- Labour and birth
- Postnatal care
- Pregnancy loss or complications (less than 20 weeks)
- Pregnancy loss (more than 20 weeks)
- Other: ___________________

1. What ethnicity do you identify as? ______________
2. What is your country of birth? ________________
3. How many years have you lived in Australia? _________________
4. What is your religion? ________________________
5. What is the primary language you speak? ______________

**Interview Questions**

1. What helped you to communicate with the healthcare provider at the hospital?
2. What made it difficult to communicate with the healthcare provider at the hospital?
3. How can healthcare providers at the hospital make it easier for you to communicate with and understand them?
4. What made you feel confident and safe to ask questions and communicate at the hospital?
5. What would make you feel safer and more confident at the hospital?
6. How would you like technology or the internet to help you communicate or find information about your [pregnancy/condition]?
7. What would make it easier to use technology or the internet to communicate or find information about your [pregnancy/condition]?
8. Is there anything else you would like us to know about your experiences?

**Additional prompts**

- - Can you say more about that?
  - Can you give me an example?
  - What was that like for you?
  - You said earlier….
  - How do you think other women….?
  - Is that your experience too?
  - We have been talking about when you were…, can you tell me…?

**Positionality Statements**

YSL is a Burmese international student who has worked on research with vulnerable communities, including those in culturally and linguistically diverse populations. As a young woman passionate about mental health, her lived experiences navigating healthcare systems have shaped her understanding of the barriers faced by Burmese women, particularly refugees, in accessing healthcare in Australia.

MM is an Iranian PhD student in Australia, researching privacy and security in healthcare data. Her background as a Farsi-speaking immigrant facilitated communication with women, encouraging them to share their experiences. It also informed her perspective on how this research could benefit others by enhancing cross-cultural communication in hospitals. Her focus on ethical data handling likely deepened her understanding of women’s concerns about having a friendly and trusted interpreter or healthcare provider with whom they can confidently share their information.

TKAN is an interpreter who undertakes interpreting at various hospitals including the study setting. She joined the project as she is committed to improving community healthcare quality in general and communication in this area in particular. Working as an interpreter as her daily work gives her the opportunity to understand some of the challenges facing the health care system, especially those experienced by the overloading at many public hospitals. As a migrant to Australia, she understands the difficulties of those in her community who have language barriers, especially for women who need treatment for their women’s health issues in a hospital setting.

Originally from Iraq and a native Arabic speaker, SG was instrumental in developing Arabic-language research materials, and conducted all interviews with Arabic-speaking women, creating a respectful and culturally sensitive environment that encouraged open dialogue. This was facilitated by her insider stance within the Arab community.

GG, who led the project design and conduct, held an outsider stance regarding the experiences of women with low English proficiency and an insider stance as a clinician with nursing experience at one of the study sites. This insider knowledge of the health service helped her to explore women’s experiences during data collection. She maintained an awareness of this positionality through structured team reflexivity discussions.

Another research team member brought her experience as a researcher and data analyst to the project. As a native Mandarin speaker from China, she had firsthand cross-cultural communication experiences at the health service in Australia. She assisted with the design of recruitment materials, conducted all interviews with Mandarin-speaking participants and conducted data analysis. As an insider, she promoted participant recruitment, streamlining the process and ensuring a comfortable environment for women to share their experiences during phone interviews.

**Supporting Quotes**

The supporting quotes presented in the manuscript are presented in the following table in their source language alongside the English translation where applicable.

**Table 1.** Supporting quotes and translations

| **Theme and subtheme** | **Supporting quotes (source language)** | **Support quotes (English translation)** |
| --- | --- | --- |
| **Overarching theme. Health is too important not to understand** | |  |
|  | من از وچود مترجم و داشتن آن مطلع نبودم و به من پیشنهاد شد. اما من این پیشنهاد را پذیرفتم چون فکر میکردم که واژه هایی وجود دارد که دکتر ممکن است بگویید و من به درستی متوجه منظور او نوشم و اطلاعات مهمی را درمورد بارداری خود از دست بدهم. درنتیجه استفاده از یک مترجم به من احساس ارامش میداد | *…there are words that the doctor may say and I would not understand them correctly and would lose important information about my pregnancy* (P4^F,M^) |
| **Theme 1. The role of interpretation** | |  |
| **Subtheme 1.1 Interpreters could facilitate mutual communication** | *When in-person, if I'm confused with something or I don't quite understand something, I can ask the interpreter at once and so the interpreter can explain it to me immediately. Therefore, it creates a connection between the interpreter and me.* (P12^B,M^) |  |
|  | *توانایی من در خواندن و نوشتن به زبان انگلیسی خوب است و میتوانم امور روزمره خود را به راحتی به تنهایی انجام دهم با این وجود در زمان مراجعه چون اطمینان نداشتم که اصطلاحات پزشکی را به زبان انگلیسی به صورت کامل متوجه میشوم یا خیر به من مترجم پیشنهاد شد و من هم قبول کردم چون نیاز داشتم اطلاعات دقیقی برای جراحی که نیاز داشتم از پزشک خود دریافت کنم*  (P39^F,G^) | *My ability to read and write English is good, and I can do daily things easily by myself. However, at the time of the visit, an interpreter was offered to me. I accepted it because I was not sure whether I would fully understand medical terms in English, and I needed to receive accurate information about the surgery that I needed, from my doctor.*  (P39^F,G^) |
| **Subtheme 1.2 Communication was hindered by inconsistent quality** | *翻译得不是很确切。然后再来是可能那个当midwife来跟我解释一些事情的时候我可能有一些疑问，然后她那个口译员，她会用她的自身经验回答我。*(Eva^M,M^) | *…the translation wasn't very accurate. Also, when the midwife was explaining things to me and I had some questions, the interpreter would answer using her own experiences.* (Eva^M,M^) |
|  | *......不受时间限制，预约通常会延迟，口译员经常在我还没结束和医护的沟通后，就到时间必须离开了。*(P67^M,M^) | *… many times, interpreters had to leave when their booking time was over, even though my consultation with the medical professionals had not completed yet.* (P67^M,M^) |
|  | *嗯，他要我签那个催产前的同意书......然后是一个电话翻译的，一个男生，但是我觉得他翻译的就是我觉得他都可能不太会翻译这种这种比较专业的这种，尤其是生产类方面的东西......嗯，然后那个翻译翻译的还没有我听的听的就是明白。*(Helen^M,M^) | *…they asked me to sign the consent form for induction… A male interpreter [was connected by telephone], but the interpreting he did, I think he probably did not know how to interpret some of the professional information, especially the things about labour and delivery… I think I understood more listening to the English. His understanding was probably no better than mine* (Helen^M,M^) |
| **Subtheme 1.3 Inaccurate communication when the interpreter was absent** | *......除了用手机*[翻译应用]*，我没有其他方式...... 不可以，比较麻烦。 就耽误医生很多的时间，而且翻译有的时候也不正确......* (Kitty^M,M^) | *...besides using my phone, I didn't have any other methods [to interpret]… it wasn't effective. It was quite inconvenient, as it took up a lot of the doctor's time, and the translation wasn't always accurate…* (Kitty^M,M^) |
|  | *对急诊是没有翻译的，医院。…...我觉得可能我也没有，我没有完全的表达嘛，因为这个东西我也不知道怎么表达。医生说没事儿，那我就就就就听呗。*(Helen^M,M^) | *No, there wasn't any interpreter at the [emergency department]… I think I did not fully express myself on that occasion, and with regard to this issue, I did not know how to say it… Since the doctors here said it was all good, so I just, just listen and accept* (Helen^M,M^) |
|  | *သူပြောတာနားမလည်တော့ ignore လုပ်လိုက်တယ်။နားမလည်တော့မသိဘူးI* (Khine^B,G^) | *Since I didn’t understand what she was saying, I just ignored it* (Khine^B,G^) |
|  | *...她说要给我打一针什么什么，我就有一点懵，没听明白，我说OK，OK，打了。对，我不知道她给我打的是。*(Helen^M,M^) | *…[the midwife] said she needed to give me a shot of something, I just, I went a bit lost, didn't really understand. I just replied. OK, OK. And [she] gave me the shot. Yeah, I did not know what she gave me* (Helen^M,M^) |
| **Theme 2. Navigating the health service with uncertainty** | |  |
| **Subtheme 2.1 Not knowing what to expect of the health service** | *…I just take my referral from my GP and to see the hospital... without any knowledge about what we will do next yeah. So that’s make me a little bit worried…* (Lily^M,M^) |  |
|  | *担心付费[口译员]* (P40^M,G^) | *[I was] concerned that [an interpreter] might involve fees* (P40^M,G^) |
|  | *، لا، ما. م ا. م ا بيكون. ما بيكون م ا بطلبولي، ما ف ي هاي الخدمة ، ما بعرف إذ ا ها ي الخدمة موجود ة عندهم ولاemergency يعني أنا سألت مر ة حكولي إن و إحن ا ما فينا نقدملك هذه الخدمة، أنا كان عندي*  (Dalal^A,G^) | *I asked once [for an interpreter], and they told me that we can't provide you with this service* (Dalal^A,G^) |
| **Subtheme 2.2 Information provided was valued but limited** | ب*عتقد إنه هذا الشيء، لا، مش منيح، لأنه أنا بضطر إني أنا أعطيها لحد يقرأ لي إياها...لأنه أنا عشان هكذا أنا بطلب مترجم لإنه أنا اللغة تابعتي مش منيحة... فياريت كمان لو يحسنوها هاي الشغلة انو يعرفوا مثلا أنه هادا عربي بحتاج لمترجم يبعثوا له الرسالة... لأنها عربية عشان ما تروح عليه الموعد*  (Dalal^A,G^) | *...it's not good, because I have to give it to someone to read it to me... they know, for example, that this is an Arab who needs an interpreter* [so] *send them the letter...* [in] *Arabic so that they don't miss the appointment...*  (Dalal^A,G^) |
|  | *但是我还是希望医院可以就是，比如说每次预约我都需要见谁，然后大概整个过程需要多长时间，就是有个大概的信息，就是如果医院信息化做得再好一点就好了*。(Mavis^M,M^) | *...I still hope the hospital could, for example, provide information on whom I need to see at each appointment and roughly how long the whole process will take. It would be better if the hospital could improve its information system.* (Mavis^M,M^) |
|  | *有啊，医院有给一个册子，有点像是……里面有第几周会有肝怎么变化，然后需要做哪些检查，对。…...但是我觉得呃，我面临的问题可能不止那些，而且说实在，嗯，我也不知道诶，就是我觉得我怀孕的时候，那时候很累，什么都不想做。...…然后有时候会翻一下那个，翻个几页看个，有可能是英文文字对我来 讲，毕竟不是我的第一语言，所以也没有什么动力去看。*(Eva^M,M^) | *…the hospital gave me a booklet. It contains details about how things develop week by week and which tests are needed… I just felt very tired when I was pregnant…Sometimes I’d flip through a few pages, but since English, after all, is not my first language, I wasn’t much motivated to read it anyway.* (Eva^M,M^) |
| **Subtheme 2.3 Women sought to supplement information provided** | *... if there some... website that midwife give to us, or some book that midwife give to me... I can trust to follow it* (Lily^M,M^) |  |
|  | *......第二个然后就是说呃中英文对应的就是说*[外部产前课]*......他就会让你更加的了解澳洲这边生产的一个过程和环境是什么样的。*(Helen^M,M^) | *...both Chinese and English were provided [in the external antenatal class], and she... offered more information for me to understand how the delivery process unfolds in Australia, and what the environment was like in an Australian hospital.* (Helen^M,M^) |
| **Theme 3. A foundation of trust** | |  |
| **Subtheme 3.1 Effective communication promoted comfort** | *相信医院的服务* (P66^M,M^) | *trust the services provided in hospitals* (P66^M,M^) |
|  | *嗯，这边医生很重视也很专业，也挺耐心的。 所以我觉得我可以很放心的跟他沟通。*(Kitty^M,M^) | *the doctors here are very attentive, professional, and patient. So I feel comfortable communicating with them*(Kitty^M,M^) |
|  | *…if we can just meet the similar* [same] *midwife, that would be really helpful… he will just know how to communicate with me, or [if they need] to speak a little bit slow down so I quite understand it…* (Lily^M,M^) |  |
|  | *Xong rồi mấy cô ấy cũng quan tâm hỏi, mỗi khi gặp đều hỏi là vấn đề tinh thần của mình có tốt hay không? Có cần hỗ trợ gì không? Có cảm thấy an toàn hay không?... Mới đầu em thấy hơi lạ lẫm. Nhưng mà rồi em cũng cảm thấy an tâm hơn.* (Jade^V,M^) | *…they also asked with care, they asked every time they met me, that if I was mentally well? Did I need any support? Did I feel safe?... At first, I felt a little strange. But then I felt more assured.* (Jade^V,M^) |
|  | *جون نميخوام برام اتفاقي بيفتد بعد بي تجربه بودنشون رو بندازن به من و بكن زبان نفهميدي*  (P61^F,M^) | *…I do not want something to happen to me and then [the HCPs] blame me for their inexperience and say that you do not understand the language.* (P61^F,M^) |
| **Subtheme 3. A person alongside supported communication and navigation** | *然后你说他们，提议如果更好的话肯定是他们如果有时间，比如说验血啊，走各个部门去检查呀，有的时候我不太认识这个地方，最好有专人带去更好，有护士带一下去验血啊或者做其他的检查也是挺好的，在医院里面。*(Kitty^M,M^) | *If I were to suggest an improvement, it would be having someone accompany me when I go for things like blood tests or check-ups in other departments...* (Kitty^M,M^) |
|  | *တစ်ခါတလေကျရင် ခိုင် ရဲ့ support worker တွေ case worker တွေကိုပြန်မေးရတယ်။ သူတို့က သူတို့ experience တွေ သူတို့သိတဲ့ဟာတွေကို ပြန်ပြောပြတယ်။ အဲ့ကျမှ သူတို့က ဘေးကနေ ဝိုင်းကူမေးပေး ပြောပေးတယ်။ ထောက်ပေးတယ်။]* (Khine^B,G^) | *[My support workers] share their experiences and what they know with me, offering guidance and helping me.* (Khine^B,G^) |
|  | *…I really liked [the interpreter] because she… had two babies and she gave me lots of the experience what she said and lots of the details about that hospital, to tell tell me not only okay this time, what to do, and also let me a little bit clear about if I meet this situation, what I maybe say. So she gave me lots of information that’s really helpful.* (Lily^M,M^) |  |
